# Adipose Tissue Dendritic Cells Enhances Inflammation by Prompting the Generation of Th17 Cells

**DOI:** 10.1371/journal.pone.0092450

**Published:** 2014-03-18

**Authors:** Yanhong Chen, Jie Tian, Xinyu Tian, Xinyi Tang, Ke Rui, Jia Tong, Liwei Lu, Huaxi Xu, Shengjun Wang

**Affiliations:** 1 Department of Laboratory Medicine, The Affiliated People's Hospital, Jiangsu University, Zhenjiang, China; 2 Institute of Laboratory Medicine, Jiangsu University School of Medical Science and Laboratory Medicine, Zhenjiang, China; 3 Department of Pathology and Centre of Infection and Immunology, The University of Hong Kong, Hong Kong, China; Université Libre de Bruxelles, Belgium

## Abstract

**Background:**

Obesity has become a global challenge for public health. It has been reported that obesity is associated with chronic inflammation. However, the mechanism for the chronic inflammation contributes to obesity remains elusive.

**Methodology/Principal Findings:**

In our study, we found a novel CD11c^+^ dendritic cell subset existed in murine adipose tissues which was immature phenotype. Moreover, as compared to the lean controls, the number of CD11c^+^ DCs and CD4^+^IL-17^+^T cells were higher in adipose tissue of high fat diet (HFD) mice. Adipose tissues derived dendritic cells (ATDCs) displayed lower levels of CD40, CD80, CD86, MHCI and MHCII expression than splenic DCs (SPDCs). However, ATDCs showed higher levels of IL-6, TGF-β and IL-23 secretion. Moreover, our *in vitro* experiments demonstrated that ATDCs were capable of promoting Th17 cell generation.

**Conclusions/Significance:**

Our results indicate the existence of CD11c^+^ DCs in adipose tissues, which displays an immature phenotype but possessing pro-inflammatory function.

## Introduction

In recent years, obesity has become a global challenge for public health since its associated metabolic abnormalities such as insulin resistance, high blood pressure and hyperglycemia are involved in the pathogenesis of type 2 diabetes and cardiovascular diseases [1]–[4]. There is increasing evidence indicating that obesity is closely related to chronic inflammation with obese adipose tissue showing some hallmarks of chronic inflammation [5], [6]; Nishimura and other authors found that large numbers of CD8^+^ effector T cells and macrophages infiltrated into high fat diet (HFD) mice adipose tissues, whereas the numbers of regulatory and CD4^+^ helper T cells were diminished [5]–[8]. Further, adipose tissues can secrete a large number of bioactive substances or adipocytokines, such as adiponectin, leptin and other pro-inflammatory cytokines as MCP-1, SDF-1α and IFN-γ. Thus, the unbalanced production of anti- or pro-inflammatory cytokines may be involved in the inflammatory process [7], [9]–[12]. However, the exact relationship between obesity and obese adipose tissue inflammation has remained partially understood.

CD11c, a member of the β_2_- integrins, has been used as an activation marker for monocytes/macrophages [13]. Furthermore, CD11c is also expressed on mouse DCs and a subpopulation of mouse monocytes/macrophages [14]. Initial studies have reported that the number of CD11c^+^ cells is significantly increased in obesity mice adipose tissues [15], [16], However, little is known about the sequence of events that lead to the infiltration of CD11c cells, and it has been unclear about CD11c^+^ cell type and the functional role in adipose tissue inflammation. Recently, accumulation of other immune pro-inflammatory mediators, such as IL-17, has been documented in adipose tissue and peripheral blood in diet-induced obesity when compared with lean controls [17], [18]. It has been reported that when co-cultured with T cells, DCs can induce the production of IFN-γ, IL-2 and, in particular, IL-17, suggesting a Th1/Th17 response [19], [20]. T lymphocytes are known to interact with DCs and regulate the inflammatory cascade [21]. Therefore, we assume that there might be a link between adipose tissue dendritic cells (ATDCs) and obesity adipose tissue inflammation.

In this study, we first detected increased number of ATDCs with an immature phenotype in HFD mice adipose tissues. Moreover, we also found that the immature ATDCs expressed higher levels of IL-6, TGF-β and IL-23, resulting in the enhancement of Th17 cells response, and then prompt diet-induced obesity adipose tissue inflammation.

## Results

### Increased number of ATDCs existed in obesity adipose tissues

**Figure 1:**
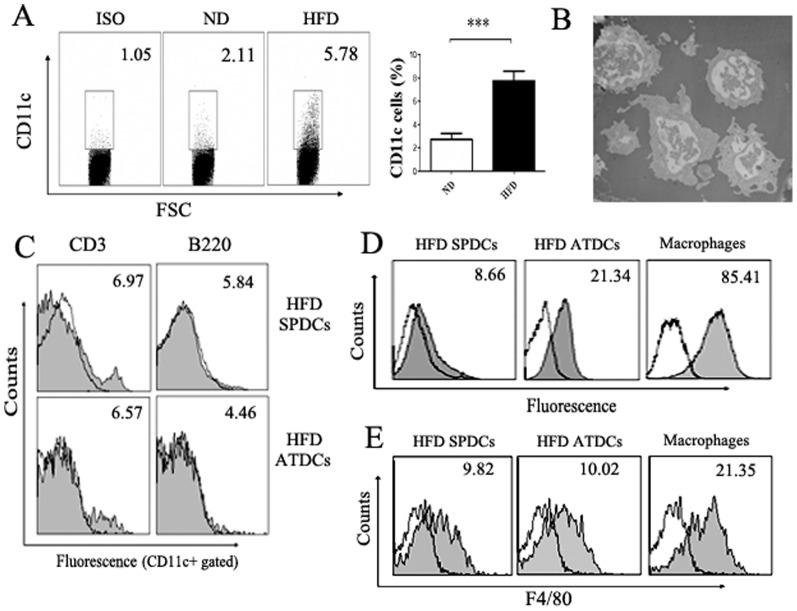
There are more DCs in HFD mice AT than ND mice AT. (A) CD11c^+^ DCs expression in HFD mice AT. CD11c^+^ DCs in HFD mice AT were analyzed using flow cytometry. And proportions of CD11c^+^ DC in the AT of HFD mice (n = 6) and ND mice (n = 6). (B) Morphology of DC-like cells in HFD mice AT under transmission electron microscope. (C) The CD11c^+^ DCs in HFD mice AT were negative for CD3 and B220. Open histograms indicate isotype control, numbers in histograms represent the geometric mean fluorescence. (D, E) The CD11c^+^ DCs in HFD mice AT are different from macrophages. (D) The phagocytosis ability of ATDCs and macrophages assessed by incubating with HcRed-expressed *E.coli* at 37°C for 4 hours. Numbers in histograms represent the geometric mean fluorescence. (E) The CD11c^+^ DCs and macrophages from HFD mice spleen or adipose tissues were stained with specific mAb against F4/80, cells were gated on the CD11c^+^ population, and numbers in histograms represent the geometric mean fluorescence of the gated cells. All data are shown as the mean ± SD of 6 samples pooled from three independent experiments. Student's t-test was used for the statistical analysis. ***p<0.001.

Using flow cytometry (FCM) analysis, CD11c^+^ cells were examined in HFD and normal diet (ND) mice adipose tissues. As shown in Fig. 1A, the frequency of CD11c^+^ cells were found significantly higher in HFD mice adipose tissues than in lean controls. As revealed by transmission electron microscopy, these CD11c^+^ cells had long dendrites, eccentric nuclei and rare lysosomes in cytoplasm, showing morphological features similar to conventional DCs (Fig. 1B). Furthermore, similar to spleen derived DCs (SPDCs), CD11c^+^ cells were negative for CD3, B220 (Fig. 1C). It is well-known that macrophages have stronger phagocytosis ability and express higher levels of F4/80 than dendritic cells, phagocytosis assay and FCM analysis indicated that the CD11c^+^ cell type that infiltrated into adipose tissues were dendritic cells rather than macrophages (Fig. 1D and E). Collectively, these data demonstrated that the CD11c^+^ cell type infiltrated into adipose tissues is ATDCs rather than macrophages and that more ATDCs were presented in obesity adipose tissues than lean control.

### ATDCs infiltrated into adipose tissues are immature DCs

**Figure 2:**
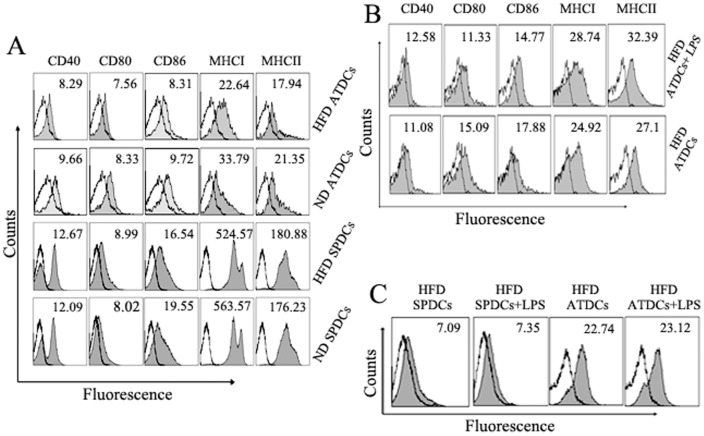
The new and special phenotype of DC cells in AT of ND and HFD mice. (A) The DCs that infiltrated into AT are immature DCs. Multicolor FCM analyses were performed on single cell suspensions that were stained with specific mAb against FITC-CD11c and PE-CD40, CD80, CD86 MHCI and MHCII, cells were gated on the CD11c population. Numbers in histograms represent the geometric mean fluorescence of the gated cells. Open histograms indicate isotype control. (B) The ATDCs of HFD mice have the relatively stable phenotype with or without LPS stimulation. The ATDCs were incubated with or without LPS (100 ng/ml) stimulation at 37°C for 12 hours and stained with specific mAb against FITC-CD11c and PE-CD40, CD80, CD86, MHCI and MHCII. Numbers in histograms represent the geometric mean fluorescence of the gated CD11c cells. (C) The ATDCs of HFD mice have the relatively phagocytosis ability with or without LPS stimulation. The ATDCs were incubated with or without LPS stimulation and HcRed-expressed *E.coli* at 37°C for 4 hours. Data are representative of three independent experiments.

To characterize the phenotype of DCs that infiltrated into the adipose tissue, FCM analysis was used to examine ATDCs in HFD and ND mice. The ATDCs isolated from HFD and ND mice expressed lower levels of CD40, CD80, CD86, MHCI and MHCII than those in SPDCs (Fig. 2A); In addition, the phagocytosis assay showed that ATDCs had more potent phagocytic capacity than SPDCs (Fig. 1D). All together, the results showed that ATDCs had characteristics of immature DCs. As known, LPS has been implicated in the maturation of immature DCs. In Figure 2B and C, the high doses (100 ng/ml) of LPS could not affect low expression of CD40, CD80, CD86, MHCI and MHCII and the phagocytosis ability of ATDCs, indicating that ATDCs had a relatively stable phenotype with or without LPS stimulation. Thus, these findings suggest that ATDCs are immature ones and have relatively stable phenotype.

### ATDCs express higher levels of IL-6, TGF-β and IL-23 than SPDCs

**Figure 3:**
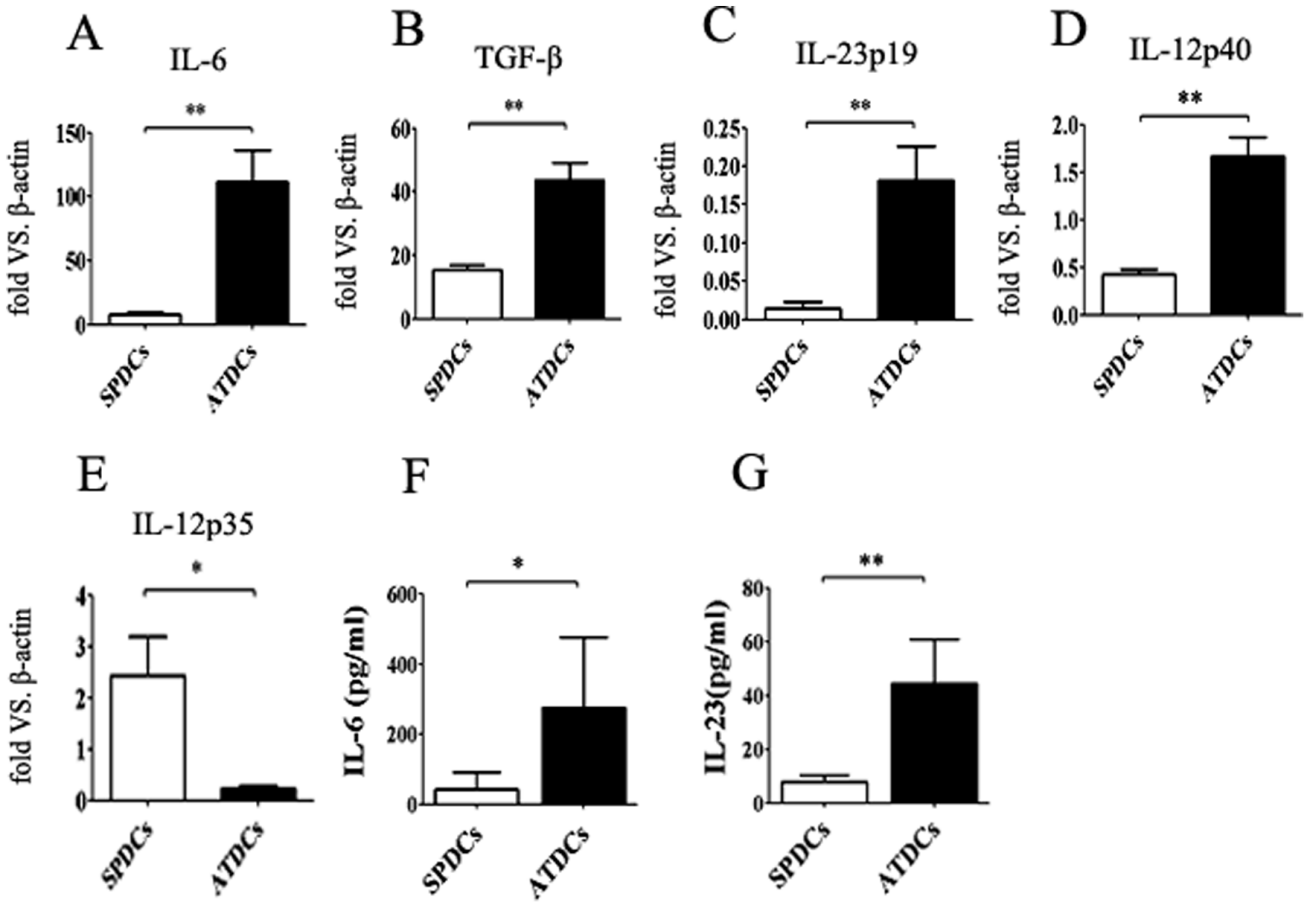
The ATDCs expressed higher levels of IL-6, TGF-β and IL-23 than SPDCs. The ATDCs and SPDCs of HFD mice were isolated by microbeads method, and then incubated them with PMA and ionomycin at 37°C for 4 hours. mRNA levels of IL-6, TGF-β, IL-23p19, IL-12p40 and IL-12p35 in the ATDCs and SPDCs were examined by qRT-PCR method. The concentration of IL-6, IL-23 in the supernatant were examined by ELISA method. Data are presented as mean ± SD of 6 samples pooled from three independent experiments. Student's t-test was used for the statistical analysis. **p<0.01, *p<0.05.

To investigate the potential function of ATDCs, the CD11c^+^ ATDCs and SPDCs were isolated by micro-beads and cultured them with PMA and ionomycin at 37°C for 4 hours and then the cytokines production were measured by quantitative real-time PCR (qRT-PCR). As shown in figure 3, we found an increase in the mRNA levels of both IL-6 (Fig. 3A) and TGF-β (Fig. 3B) of HFD mice ATDCs than SPDCs. In addition, other cytokines such as IL-23p19, IL-12p40 and IL-12p35 mRNA levels were also measured in ATDCs and SPDCs, and found that the ATDCs expressed higher levels of IL-23p19 (Fig. 3C), and IL-12p40 (Fig. 3D) mRNA than SPDCs. Surprisingly, the IL-12p35 (Fig. 3E) mRNA in the ATDCs was hardly to be found. As IL-6 and IL-23 are the important IL-17-inducing cytokines, these findings suggested that ATDCs can lead to some pro-inflammatory mediators such as Th17 cells trafficking into adipose tissue in HFD mice. The supernatant was also collected to measure IL-6 and IL-23 by ELISA. As indicated in Fig. 3G and F and in agreement with our previous results, ATDCs expressed higher levels of IL-6 and IL-23 than SPDCs.

### Higher level of pro-inflammatory mediator Th17 cells was existed in obesity adipose tissues

**Figure 4:**
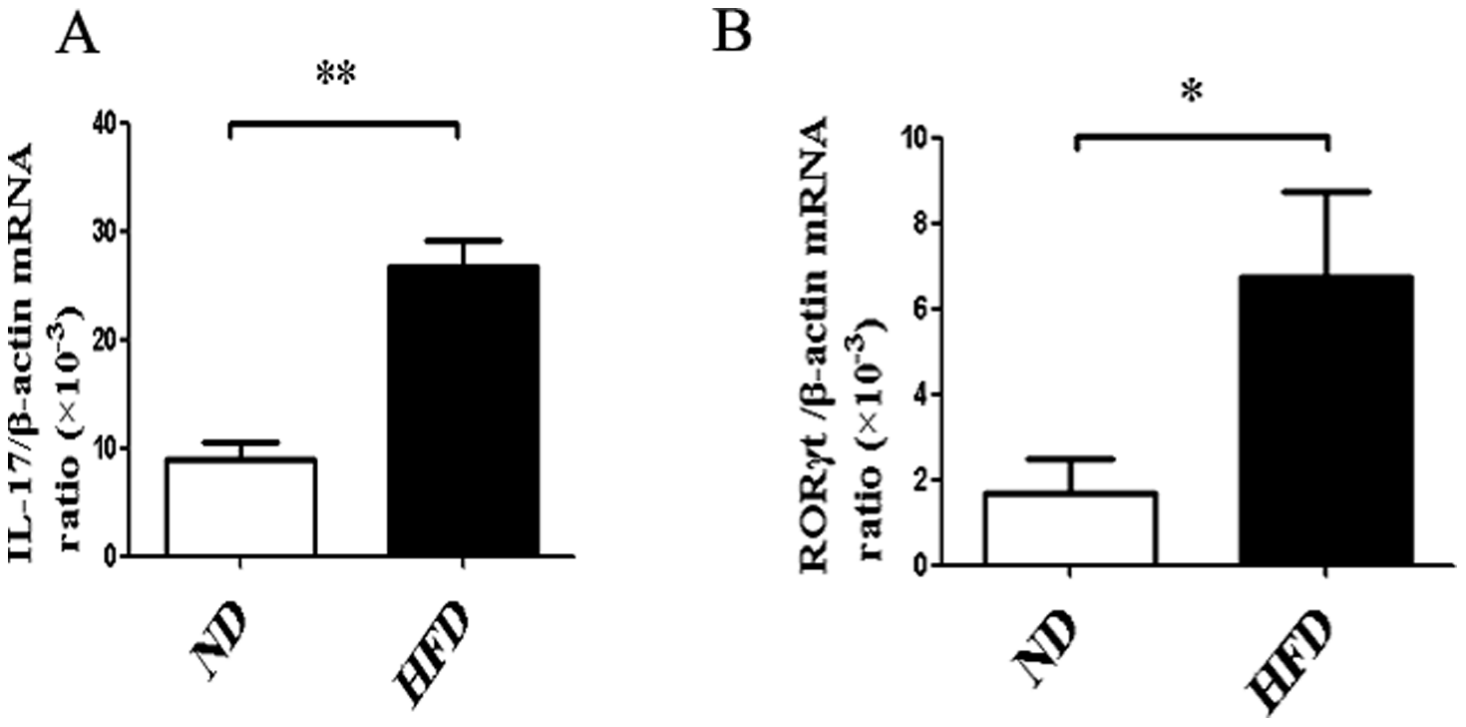
Higher level of Th17 cells were existed in obesity adipose tissues. (A) The CD4^+^T cells of HFD and ND mice AT were isolated by microbeads method and then incubated them with PMA and ionomycin at 37°C for 4 hours. mRNA levels of IL-17 in the HFD and ND mice AT were examined by qRT-PCR method. (B) mRNA levels of RORγt in the HFD and ND mice AT were examined by qRT-PCR method. Data are presented as mean ± SD of 3 samples pooled from three independent experiments. Student's t-test was used for the statistical analysis. **p<0.01, *p<0.05.

Previous studies have shown that obesity is linked not only to diabetes and heart diseases [22], but also to the increased incidence of inflammatory diseases such as multiple sclerosis in human [23], psoriasis [24] and experimental autoimmune encephalomyelitis in mice [25]. It is evident that, obesity is associated with a state of low-grade chronic inflammation [26], there are also some studies reported that the pro-inflammatory cytokine, IL-17, is expressed in higher level in HFD mice adipose tissues than ND mice [18]. In our study, to investigate whether diet-induced obesity can attract more CD4^+^IL-17^+^ T cells into adipose tissues, the CD4^+^T cells from HFD and ND adipose tissues were isolated by micro-beads and cultured them with PMA and ionomycin at 37°C for 4 hours. To our delight, both CD4^+^IL-17^+^ T cells (Fig. 4A) and its specific transcription factor RORγt (Fig. 4B) showed higher levels of HFD mice adipose tissues than ND mice. Along with previous study, our work also demonstrated diet-induced obesity can attract more Th17 cells into adipose tissue which can prompt obesity adipose tissue inflammation.

### ATDCs promote the adipose tissue inflammation by enhancement of Th17 cells response

**Figure 5:**
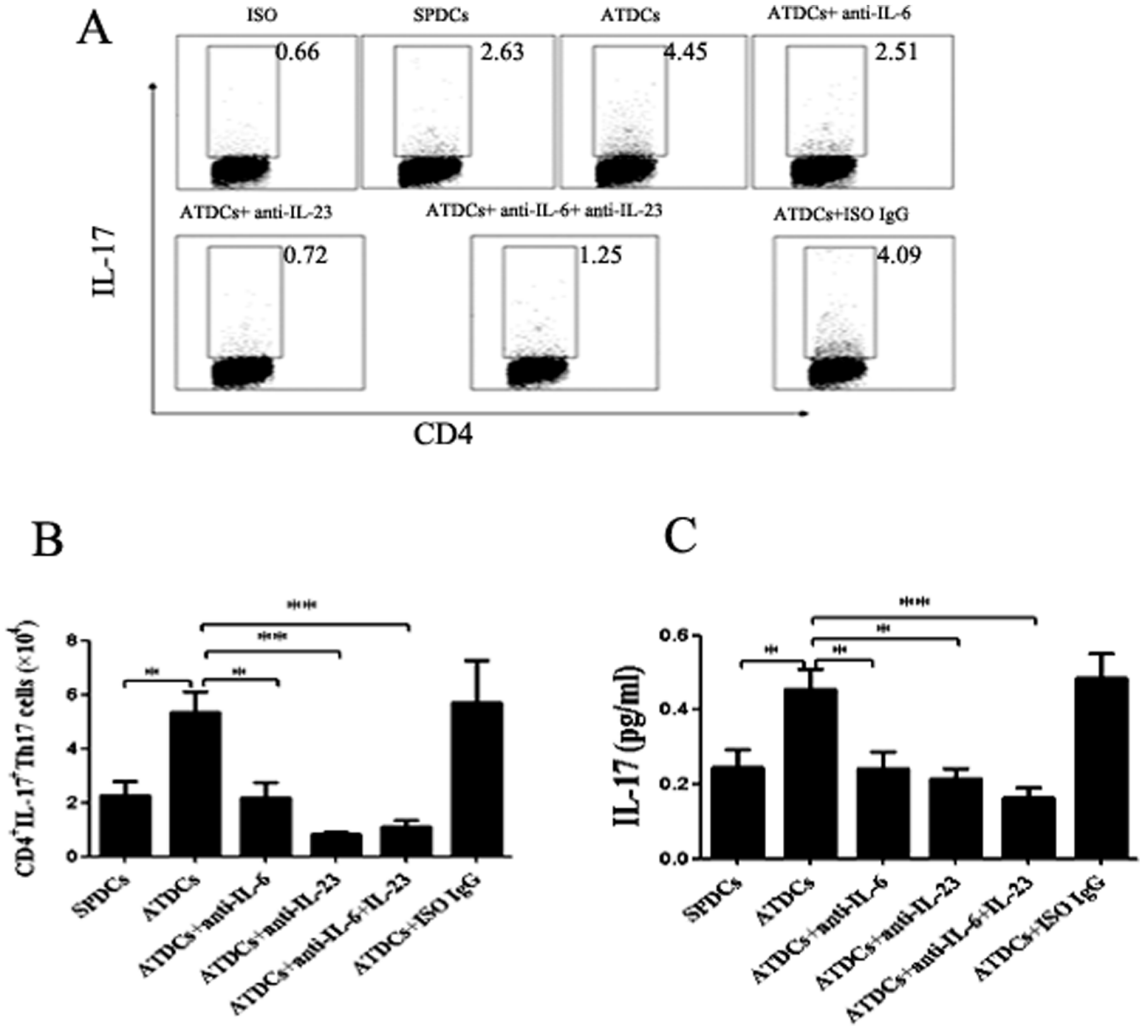
Interplay between ATDCs and CD4^+^T cells. (A,B) 2×10^5^ ATDCs or SPDCs were isolated from HFD mice AT and SP by microbeads method and co-cultured them with 1×10^6^ CD4^+^T cells that isolated from 6-8 weeks old male C57BL/6 mice for 7 days. The single cell suspensions of the co-culture system which was in the presence or absence of anti-IL-6, anti-IL-23 or isotype IgG were stained with specific mAb against FITC-CD4 and PE-IL-17. And the multicolor FCM analyses were performed on the single cell suspensions. (C) The concentration of IL-17 in the supernatant were examined by ELISA method. Data are presented as mean ± SD of 3 samples pooled from three independent experiments. Student's t-test was used for the statistical analysis. **p<0.01, *p<0.05.

From the above data, we assume that there may be a link between ATDCs and obesity adipose tissue inflammation which caused by Th17 cells and then analyzed the cellular interplay by which inflammation develops in HFD mice adipose tissues. Noticeably, IL-6, TGF-β and IL-23 can induce distinct immune responses by prompting Th17 cell differentiation. From our previous study, we found that ATDCs can express higher levels of IL-6, TGF-β, IL-23 (Fig. 3). Therefore, we hypothesized that ATDCs can promote the differentiation of Th17 cells from CD4^+^T cells. To test this hypothesis, we co-cultured splenetic CD4^+^T cells with ATDCs or SPDCs to determine whether the ATDCs can prompt the Th17 cells response. Results clearly indicates that, when co-cultured with ATDCs, the percentage of CD4^+^IL-17^+^T cells were significantly increased than with SPDCs, and the Th17 cell numbers were also higher when co-cultured with ATDCs than SPDCs (Fig. 5A and B). The supernatant was also collected to measure IL-17 by ELISA (Fig. 5C). Our results also clearly show that, the percentage of Th17 cells and the Th17 cell numbers can be decreased by anti-IL-6 and/or anti-IL-23 antibodies in this co-culture system. Taken together, our results support that ATDCs can prompt the enhancement of Th17 cells response.

### HFD mice adipose tissues can express higher level of ATDCs chemo-attractant CCL20

**Figure 6:**
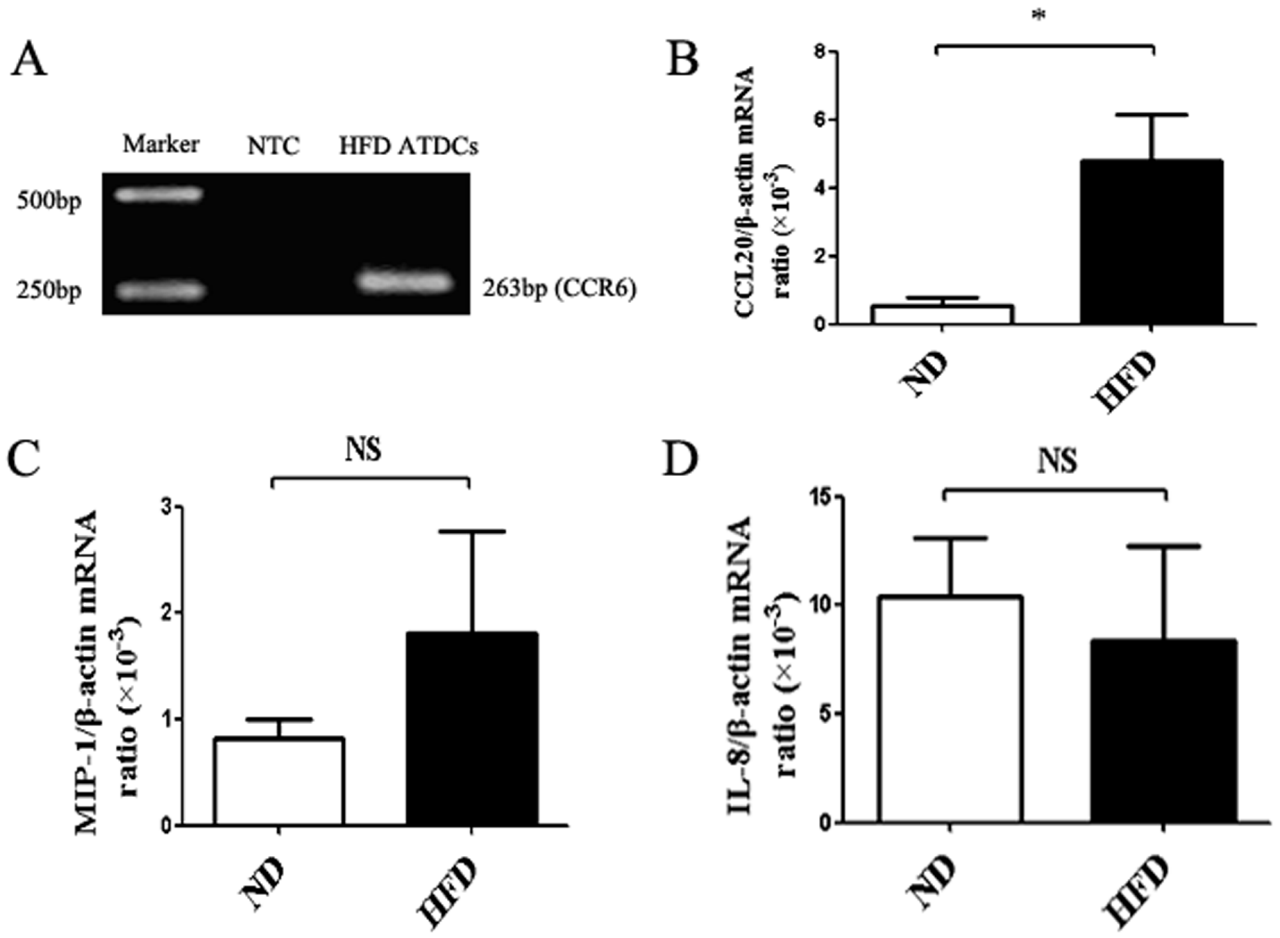
HFD mice AT expresses higher levers of ATDCs chemo-attractant CCL20 than ND mice. (A) mRNA levels of CCR6 were determined by RT-PCR method. mRNA levels of CCL20(B), MIP-1(C) and IL-8(D) were determined by qRT- PCR method on HFD mice AT and ND mice AT relative to β-actin. Data are presented as mean ± SD of 5 samples pooled from three independent experiments. Student's t-test was used for the statistical analysis. *p<0.05, N.S no significance.

To assess why adipose tissue attracted ATDCs, the ATDCs gene expression was analyzed. As expected, strong positive signals of CCR6 (Fig. 6A) were detected. Notably, the chemo-attractant CCL20 receptor is CCR6, and to further characterize the ATDCs chemo-attractant CCL20, real-time PCR analysis were performed on HFD and ND mice adipose tissues. The level of CCL20 transcripts of HFD mice adipose tissues is higher than ND mice adipose tissues (Fig. 6B). However, we did not find other chemo-attractants such as MIP-1 and IL-8 higher compared to lean controls (Fig. 6C and D).

## Discussion

Over the past several decades, type 2 diabetes and other metabolic syndrome have increased at an alarming rate and obesity increases the risk for type 2 diabetes and other metabolic syndrome such as cardiovascular diseases [27]–[30]. Recently, it has been reported that large numbers of CD8^+^ effector T cells and pro-inflammatory macrophages infiltrated into HFD mice adipose tissues, whereas the numbers of regulatory and CD4^+^ helper T cells were diminished [5]–[8]. That means obese adipose tissues shows some typical symptoms of chronic inflammation, leading to systemic insulin resistance and type 2 diabetes and other cardiovascular diseases [8]. So it is very important to reveal the mechanism of obese adipose tissue inflammation.

To date, there is no evidence can explain the relationship between obesity and adipose tissue chronic inflammation. Wu and his partners found that the expression of CD11c^+^ cells is higher in HFD mice adipose tissues than ND mice, but they didn't demonstrate the phenotype and function of the CD11c^+^ cells. There were also some other groups indicated that HFD mice adipose tissues express higher level of IL-17 [18], but unfortunately, they didn't show clearly where the IL-17 comes from. More important, we report, for the first time to our knowledge, there may be a link between CD11c^+^ DCs and obese adipose tissue inflammation which caused by Th17 cells.

In this study, we confirmed that the CD11c^+^ dendritic cells phenotype, which was F4/80 low, was increased in the adipose tissues of HFD mice than ND mice, we also report herein our novel finding that in HFD mice adipose tissues, the infiltrated ATDCs were immature ones, obtaining stronger phagocytosis ability and still have stable phenotype with or without LPS stimulation, and another innovative finding was that the ATDCs can prompt the Th17 cells response as ATDCs express higher levels of IL-6, TGF-β and IL-23. Meanwhile the CD4^+^T cells were isolated from HFD and ND mice adipose tissues, and the CD4^+^IL-17^+^ T cells were also increased in HFD mice adipose tissues compared with ND mice. Along with previous studies that obesity is associated with chronic low-grade inflammation, as demonstrated by increased levels of pro-inflammatory mediators such as Th17 cells [26].

With the increased levels of CD8^+^T cells and macrophages, we found there was an increased level of CD11c^+^ DCs in adipose tissues of HFD mice than ND mice. Besides, factor(s) in adipose microenvironment which can attract the immature ATDCs into adipose tissues needs to be further studies. One factor worth mentioning may be special antigen stimulation, as suggested by their unusual high state of activation and the imprint of anti-genetic selection of obesity ATDCs. Depending on such special antigen(s), they could stimulate circulating DCs, making them to exit the lymph nodes or spleens and invade the re-stimulated ATDCs filtering through obesity adipose tissues and thereby prompting adipose tissue inflammation. A second factor is almost certainly chemokines, given the unique pattern of chemokine–chemokine receptor gene expression by the ATDCs isolated from adipose tissues. In our work, we found that HFD mice adipose tissues can express higher level of CCL20 than ND mice adipose tissues and the CCL20 receptor CCR6 is also expressed in ATDCs; However,we did not find other chemokines express in higher levels in HFD mice adipose tissues than in ND mice. Finally, in HFD mice adipose microenvironment, there may be a role for adipokines in nurturing ATDCs. Adipose tissue is a paracrine and endocrine organ that can secrete a large number of bioactive substances or adipocytokines such as adiponectin, leptin, MCP-1 and so on. For instance, a recent study reported the negative effect of leptin on the proliferation of Treg cells [31]. Similar to the relationship between the Treg cells and leptin, there may be other factor(s) that can also maintain ATDCs in obesity adipose tissues.

IL-17, produced by not only CD4^+^Th17 lymphocytes but also CD8^+^T cells, γδT cells, NK cells and monocytes, is a potent pro-inflammatory mediator. We and others [18], [32]–[36] have demonstrated that obesity is associated with increased inflammation and thus, obesity inflammation maybe homeostatic inflammation. Meanwhile, we wondered what function of ATDCs performing in obesity adipose tissue. Therefore, we first identify that there is a link between ATDCs and obesity adipose tissue inflammation which is caused by pro-inflammatory mediator Th17 cells. ATDCs express higher levels of IL-6, TGF-β, IL-23 that are essential cytokines for Th17 cells proliferation or differentiation, giving us a new explanation for obesity adipose tissue inflammation that is caused by Th17 cells. As known, IL-6 and IL-23 are important cytokines for Th17 cell proliferation. Our data demonstrated that the differentiation of Th17 cells was almost inhibited by neutralization of IL-23. In contrast, anti-IL-6 antibody did not inhibit the differentiation of Th17 cells thoroughly than anti-IL-23 antibody did. When analyzed together, these data indicate that IL-23 plays more important role in enhancing synthesis of IL-17.

In summary, our observation implicate the immature ATDCs as an important cell type whose production is increased in obesity adipose tissue than lean controls and the increased ATDCs could promote the production of pro-inflammatory Th17 cells which in turn plays a prominent role in obesity adipose tissue inflammation and also opens new avenues in the investigation of the potential connection between ATDCs, obesity adipose tissue inflammation, Th17 cell type, type 2 diabetes and cardiovascular diseases.

## Materials and Methods

### Mice

The male C57BL/6 mice (4 weeks age) were obtained and cared followed institutional guidelines under a protocol approved by Yangzhou University. To examine the time-course of changes of the cell populations in epididymal adipose tissues of diet-induced obesity, the mice were divided into two groups and fed a normal low fat chow diet (NF; 10% kcal from lard) or a high-fat diet (HF; 60% kcal from lard). All mice were allowed free access to water and food. After 18–22 weeks, the adipose tissues were removed and minced it into small pieces (∼2 mm) and then incubated them at 37°C for about 2 hours in collagenase II (Sigma-Aldrich, St. Louis, MO)) with gentle stirring. All animal experiments performed in this study were approved by the Jiangsu University Animal Ethics and Experimentation Committee.

### Cell isolation and purification

Adipose tissues of HFD or ND mice were minced and then digested with collagenaseII for 1–2 h at 37°C and then ATDCs and SPDCs were isolated from obesity adipose tissues and spleen. ATDCs or SPDCs were purified by biotin-conjugated anti-mouse CD11c monoclonal Ab (mAb) and anti-biotin micro-beads (Miltenyi Biotec GmbH, Bergisch Gladbach, Germany), according to the manufacturer's instructions. Cell viability was more than 95%.

### RNA isolation and real-time RT-PCR

To determine gene expression, total RNA was isolated not only from adipose tissues of ND or HFD mice aged 18–22 weeks but also from ATDCs or SPDCs isolated by biotin-conjugated anti-mouse CD11c monoclonal Ab (mAb) and anti-biotin micro-beads. Using the TRIzol (Invitrogen, Carlsbad, CA) isolated according to the manufacturers' instructions. To generate cDNA, isolated RNA was eluted in RNase free water and used as the template for reverse transcription. ReverTra Ace qPCR RT Kit (Toyobo, Osaka, Japan) was used to prepare cDNA according to the manufacturers' instructions. Real-time PCR was performed in duplicate using Bio-Rad SYBR green super mix (Bio-Rad, Hercules, CA). The sequences for the primers used are: CCL20, sense, 5-CGA CTG TTG CCT CTC GTA CAT-3; anti-sense, 5-AGC CCT TTT CAC CCA GTT CT-3, IL-8, sense, 5-GAA GTG GCA GAA GCT AAC CG-3; anti-sense, 5-GCT GGG ATT CAC CTC AAG AA-3, MIP-1, sense, 5-TCC CAC TTC CTG CTG TTT CTC-3; anti-sense, 5-TGC CTC TTT TGG TCA GGA ATA C-3, IL-12p19, sense, 5-GCT TGC AAA GGA TCC ACC-3; anti-sense, 5-CCA GTA GGG AGG CTA GAA G-3, IL-23p35, sense, 5-TGA CAT GGT GAA GAC GGC-3; anti-sense, 5-GCC TGG AAC TCT GTC TGG TA-3, IL-17, sense, 5-GGA CTC TCC ACC GCA ATG-3; anti-sense, 5-CAC ACC CAC CAG CAT CTT C-3, IL-6, sense, 5- GGC CTT CCC TAC TTC ACA AG-3; anti-sense, 5-ATT TCC ACG ATT TCC CAG AG-3, TGF-β, sense, 5-AAC CGG CCC TTC CTG CTC CTC AT-3; anti-sense, 5-CGC CCG GGT TGT GTT GGT TGT AGA-3, IL-12p40, sense, 5- CAC AAA GGA GGC GAG ACT C-3; anti-sense, 5- AGT CAG GGG AAC TGC TAC TG-3, CCR6, sense, 5- GAG TCC TAC TTT GGA ACG GAT G-3; anti-sense, 5-GGT AGG GTG AGG ACA AAG AGT ATG-3, RORγt, sense, 5-CCA CCA TAT TCC AAT ACC TT-3; anti-sense, 5-GCT GTC TGG ACC CTG TTC T-3. Each gene was amplified at least three times and cDNA concentration differences were normalized to β-actin with the following primers: sense, 5-TGG AAT CCT GTG GCA TCC ATG AAA C-3; anti-sense, 5-TAA AAC GCA GCT CAG TAA CAG TCC G-3.

### Flow cytometric analysis

The C57BL/6 mice at the age of 18–22 weeks were killed and removed the epididymal adipose tissues and minced it into small pieces (∼2 mm). Then the pieces of adipose tissues were collected and incubated them at 37°C for about 2 hours in collagenase II with gentle stirring. The digested tissues were centrifuged at 500 g for 5 min. The resultant pellet containing the total lymphocytes were re-suspended in PBS and filtered it through a 100 M mesh, and we finally re-suspended them in 100 μl PBS supplemented with 5% FBS. All these cells were incubated with FITC-CD11c, PE-CD40, CD80, CD86, MHCI, MHCII (eBioscience, San Diego, CA) conjugated antibodies or respective isotype controls for 30 min at 4°C. All the staining was performed according to manufacturers' protocol. Flow cytometry was performed using FACS Calibur instrument (Becton Dickinson) and WinMDI 2.8 software was used to analyze the data.

### Enzyme-linked immunosorbent assay

The ATDCs and SPDCs were isolated from HFD mice by biotin-conjugated anti-mouse CD11c monoclonal Ab (mAb) and anti-biotin micro-beads. 0.5×10^6^ DCs per ml were incubated in medium supplemented with ionomycin and PMA. After 4 hours incubation, cells were harvested for determination of levels of IL-6, TGF-β, IL-23 and IL-12 by quantitative real-time PCR (qRT-PCR). The supernatant was collected for determination of levels of IL-6 and IL-23 by ELISA according to the protocols provided by the manufactures.

### Cells co-culture assays *in vitro*

CD4^+^T cells were isolated from wild-type 6-8 week-old C57BL/6 mice spleen by biotin-conjugated anti-mouse CD4 monoclonal Ab (mAb) and anti-biotin micro-beads. ATDCs and SPDCs were also isolated from HFD 18–22 week-old C57BL/6 mice adipose tissue and spleen. In the 24-well plates, 1×10^6^ CD4^+^T cells per well containing RPMI 1640 medium with 20% FBS, 1% penicillin/streptomycin were co-cultured with 2×10^5^ ATDCs or SPDCs for 7 days, after which the cells were harvested for determination of the CD4^+^ IL-17^+^ T cell differentiation. The cells were stained with FITC-CD4, PE-IL-17 (eBioscience, San Diego, CA) conjugated antibodies or isotype controls. The supernatant was also collected for determination of levels of IL-17 by ELISA as previously described. Before co-culture, 5 μg/ml anti-CD3 is incubated overnight at 4°C.

### Statistics

Results are given as the mean ± SD. A 2-sided student t test was used to analyze the data. SPSS 11.5 software was used to perform all analysis. Differences were described as significant at a *P* value less than 0.05.
